# Application of the screening method to monitor influenza vaccine effectiveness among the elderly in Germany

**DOI:** 10.1186/s12879-015-0882-3

**Published:** 2015-03-20

**Authors:** Cornelius Remschmidt, Thorsten Rieck, Birte Bödeker, Ole Wichmann

**Affiliations:** Immunization Unit, Robert Koch Institute, Berlin, Germany; Charité – University Medicine Berlin, Berlin, Germany

**Keywords:** Influenza, Vaccine effectiveness, Screening method, Elderly

## Abstract

**Background:**

Elderly people are at increased risk for severe influenza illness and constitute therefore a major target-group for seasonal influenza vaccination in most industrialized countries. The aim of this study was to estimate influenza vaccine effectiveness (VE) among individuals aged 60+ years over three seasons and to assess if the screening method is a suitable tool to monitor influenza VE in this particular target-group in Germany.

**Methods:**

We identified laboratory-confirmed influenza cases aged 60+ years through the national communicable disease reporting system for seasons 2010/11, 2011/12 and 2012/13. Vaccination coverage (VC) data were retrieved from a database of health insurance claims representing ~85% of the total German population. We applied the screening method to calculate influenza subtype-specific VE and compared our results with VE estimates from other observational studies in Europe.

**Results:**

In total, 7,156 laboratory-confirmed influenza cases were included. VE against all influenza types ranged between 49% (95% confidence interval [CI]: 39–56) in 2011/12 and 80% (95% CI: 76-83%) in 2010/11. In 2010/11 subtype-specific VE against influenza A(H1N1)pdm and B was 76% and 84%, respectively. In the following seasons, VE against influenza A(H1N1)pdm, A(H3N2) and B was 87%, -9% , 74% (2011/12), and 74%, 39%, 73% (2012/13). VE was higher among hospitalized compared to non-hospitalized influenza A cases. Seventeen observational studies from Europe reporting subtype-specific VE among the elderly were identified for the respective seasons (all applying the test-negative design) and showed comparable subtype-specific VE estimates.

**Conclusions:**

According to our study, influenza vaccination provided moderate protection against laboratory-confirmed influenza A(H1N1)pdm and B in individuals aged 60+ but no or only little protection against A(H3N2). Higher VE among hospitalized cases might indicate higher protection against severe influenza disease. Based on the available data, the screening method allowed us to assess subtype-specific VE in hospitalized and non-hospitalized elderly persons. Since controlling for several important confounders was not possible, the applied method only provided crude VE estimates. However, given the precise VC-data and the large number of cases, the screening method provided results being in line with VE estimates from other observational studies in Europe that applied a different study design.

**Electronic supplementary material:**

The online version of this article (doi:10.1186/s12879-015-0882-3) contains supplementary material, which is available to authorized users.

## Background

Elderly people are at increased risk for severe influenza disease or influenza-associated complications [1]. Therefore, the World Health Organization (WHO) and National Immunization Technical Advisory Groups (NITAGs) in most industrialized countries recommend seasonal influenza vaccination for this particular at-risk group [2-5]. The European Union aims to achieve a vaccination coverage of >75% in this age-group to reduce influenza-associated morbidity and mortality [6]. However, due to progressive deterioration in both innate and adaptive immune function, aging is associated with a reduced immune response to influenza vaccines [7]. Although it seems plausible that this also translates into reduced vaccine effectiveness (VE) in this age-group, there are still conflicting conclusions drawn by authors in respect to influenza VE among the elderly [8]. While some authors concluded that evidence on influenza VE in this age-group is poor [8] or even lacking [9], others found – based on results from post-marketing observational studies – evidence for a substantial reduction in influenza-related morbidity and mortality among the elderly [10].

To guide national immunization strategies and research into the development of improved influenza vaccines, annual estimates of influenza VE in vaccination target-groups are important [11]. In fact, suboptimal effectiveness of seasonal influenza vaccines has recently led researchers and policy makers to advocate for better vaccines [9,12]. Since VE varies not only by characteristics of the vaccinees (e.g., age or underlying medical conditions), but also by influenza season and subtype, many countries have established annual assessments of influenza VE. An increasingly applied method to assess influenza VE is the test-negative design (TND) [13-17]. Patients with medically attended acute respiratory illness (ARI) constitute the study population, and participants are considered as cases if they are tested positive for influenza and as controls if tested negative in a specific time-period after symptom onset [18,19]. In addition to the TND, the screening method has been used to estimate seasonal and pandemic VE in various settings [20-24]. This method estimates VE by comparing vaccination coverage (VC) in influenza-positive cases with VC in the population where the cases derived from (e.g., the same age-group) [25]. If representative data on influenza cases and influenza VC are available, the screening method provides an inexpensive and ready-to-use method that can be useful in providing early VE estimates [26] or in identifying changes in VE over time when conducted repeatedly [14,20]. However, compared to TND the screening method seems more prone to selection bias, and controlling for important confounders, such as comorbidities, might not be possible due to lack of availability of these data [25]. Nonetheless, when applying stratified analyses or limiting the analysis to a more homogeneous population (such as elderly persons) the risk of bias can be reduced.

With this study we aimed to (i) estimate VE against laboratory-confirmed influenza for the seasons 2010/11- 2012/13 among persons aged 60+ years, (ii) compare VE in preventing hospitalized and non-hospitalized influenza, and (iii) assess if the screening methods is a suitable tool for the monitoring of influenza VE among the elderly in Germany.

## Methods

### Database for determining population vaccination coverage

The source for seasonal influenza VC was the central database of health insurance claims of the Associations of Statutory Health Insurance Physicians (ASHIPs) in Germany. ASHIPs administrative regions are identical to the geographic regions of the 16 federal states, except for one federal state that is divided into two ASHIP regions. ASHIPs regularly receive claims data from all ASHIP-associated physicians for ambulatory services, covering more than 85% of the total population in Germany. Within the ASHIP vaccination monitoring project, anonymous data of ASHIPs are transferred to the Robert Koch Institute (RKI) on a quarterly basis and include information on age (month/year), date of patient contact, as well as date and type of vaccination [27]. We extracted information on seasonal influenza vaccination for seasons 2010/11, 2011/12 and 2012/13 among all individuals aged 60+ years and calculated VC for the total elderly population and by age-strata. Data were available from 16 of the 17 ASHIP regions.

### Identification of influenza cases

In Germany, laboratory-confirmed influenza infections are notifiable according to the German Protection against Infection Act since 2001 [28]. We identified influenza cases through the national communicable disease reporting system. Within this system, case-based data of patients with laboratory-confirmed influenza infection are collected by local public health authorities and transmitted electronically via state health departments to RKI. Cases reports include information on age (month/year), place of residence, date of disease onset, date of notification, receipt of influenza vaccine, laboratory confirmation of disease, influenza type and subtype, and hospitalization. Additional information, such as type or name of the influenza vaccine is rarely reported and information on comorbidities is almost never provided.

For our analysis, notified cases aged 60 years and older were included if infection was laboratory-confirmed (i.e., detection of influenza antigens using enzyme immunoassays, polymerase chain reaction, or virus culture), and if information on cases’ vaccination status was available. A case was defined as vaccinated, if classified as “vaccinated in this current season” in the electronic surveillance system. An exact date of vaccination was provided for 66% of all vaccinated influenza cases between 2010/11-2012/13, of whom only 9 had a symptom onset <14 days post vaccination. These cases were considered as “non-vaccinated”. For the remaining 34% of vaccinated cases, for whom the date of vaccination was missing, the number of persons with disease onset within 14 days after vaccination was regarded as unlikely. Since this assumption can potentially increase the numbers of vaccine failures and decrease VE thereby, we conducted a sensitivity analysis by excluding people from the analysis who were vaccinated <14 days prior to symptom onset (results are presented in Additional file 1: Table S1). For each season, proportion of laboratory confirmed influenza cases vaccinated with 95% CIs stratified by age-group (60+, 60–69, 70–79, 80+ years), influenza subtype and hospitalization status (yes/no) were calculated.

The data on influenza cases reported under the German Protection against Infection Act are freely available from the national surveillance database at https://survstat.rki.de.

### Definition of influenza seasons

We included cases with disease onset during the study period that was defined from week 40 of the previous to week 20 of the following year for each of the three seasons under investigation. In a sensitivity analysis, we restricted VE calculation to cases with disease onset during the respective influenza season as defined by RKI’s Working Group for Influenza. According to the Working Group, which collects syndromic and laboratory data within a sentinel system of primary care physicians in Germany, influenza seasons were defined from weeks 50/2010-14/2011, 6/2012-16/2012 and 50/2012-16/2013, respectively [29,30].

### Statistical analysis

We used descriptive analysis for seasonal influenza VC. In brief, VCs and 95% CIs were calculated as proportion of patients having received claimed influenza vaccinations among the total statutory health insured population by region, age-group, and season.

### Calculation of vaccine effectiveness (VE)

VE and 95% CIs were calculated using the screening method according to Farrington [25] with the following formula:

$$ VE=\frac{PV-PCV}{PV\left(1-PCV\right)} $$

where PV is the proportion population vaccinated (i.e. VC) in the respective age-group and where PCV is the proportion of cases vaccinated. We calculated VE by season, age-group, hospitalization status, and influenza subtype.

### Identification of factors associated with vaccine failure

We defined vaccine failure as a laboratory-confirmed influenza infection in a person vaccinated against influenza in the respective season. To examine independent predictors of breakthrough infections (leading to vaccine failure) for each influenza season and by influenza subtype, we used a logistic regression model including potential confounding factors (age, sex, hospitalization, week since start of influenza season) and calculated odds ratios (OR) and 95% CI.

Two-sided hypothesis tests were performed and a p-value of ≤0.05 was considered statistically significant. The statistical software package STATA®, version 11 (STATA Corp., College Station, TX, USA) was used.

### Identification of observational VE studies from Europe

To compare our study results with influenza VE estimates among the elderly in other observational studies in Europe, we conducted a systematic literature search in PubMed using the following search strategy: (((influenza) AND vaccine) AND effectiveness) AND Europe (restrictions: publication year 2010 to 2015; language: English; species: human). Date of last search was 17 February 2015. In addition, we complemented our search strategy by comparing identified studies with those identified by a recently published meta-analysis by Darvishian et al. [31]. Only studies were included that reported subtype-specific VE estimates in at least one of the seasons 2010/11-2012/13. We extracted (subtype-specific) VE estimates and compared these results with ours. We excluded studies which used other designs than the TND and studies providing “early”, “mid-season”, or “interim estimates”.

### Ethical considerations and data protection

Since we analyzed (i) anonymous VC-data from ASHIP and (ii) anonymous data on influenza cases that are collected routinely within the German surveillance system for notifiable diseases, ethical approval was not obtained. The Federal Commissioner for Data Protection and Freedom of Information in Germany has approved the ASHIP vaccination monitoring project.

### Standards of reporting

We followed the STROBE guidelines in reporting this study [32].

## Results

### Influenza vaccination coverage among the elderly 2010/11-2012/13

VC by age-group and season is shown in Table 1. Over the three seasons, VC decreased slightly in all age-groups. In the age-group 60+ years, the VC assessment based on data from an average 18 million (i.e. ~85% of the total 22 million) individuals of this age-group living in Germany. VC in this age-group ranged between 37% and 44% and increased with age (Table 1).

**Table 1:** Seasonal influenza vaccination coverage by age group estimated from health insurance claims data, influenza seasons 2010/11-2012/13, Germany*

**Age-group or place of residence**	**2010/11 %**	**2011/12 %**	**2012/13 %**
≥18 years	21.0	19.8	17.4
≥60 years	44.0	42.1	37.4
60-69 years	35.9	33.0	28.2
70-79 years	48.3	46.7	42.1
≥80 years	51.4	50.6	45.6

*Upper and lower bounds of the 95% CIs were 0.2% points above or below the point estimates and are not shown in the tables.

### Notified influenza cases aged ≥60 years, 2010/11-2012/13

In total 1,371 (2010/11), 1,034 (2011/12) and 6,803 (2012/13) laboratory-confirmed influenza cases aged 60+ were notified to the RKI during the study period, respectively. The high number of laboratory-confirmed cases in the season 2012/13 reflected the severity of the influenza wave in this particular season in Germany [29]. Among all notified cases, information on laboratory confirmation was available in more than 96%, and information on vaccination status was available in more than 75%. In the final analysis we included 1,174 (85.6%) cases in the season 2010/11, 767 (74.1%) cases in the season 2011/12, and 5,217 (76.7%) cases in the season 2012/13, respectively. In two seasons (2011/12 and 2012/13), excluded cases were older than included cases and in the season 2012/13, excluded cases were more likely to be hospitalized (40% vs 44%, p = 0.01; for details see online Additional file 1: Table S2).

### Characteristics of included influenza cases aged ≥60 years

Characteristics of included influenza cases are shown in Table 2. Proportions of cases vaccinated ranged from 14% in season 2010/11 to 27% in season 2011/12. In two seasons (2010/11 and 2012/13), subtype A(H1N1)pdm was the dominant influenza A virus. The season 2011/12 was dominated by subtype A(H3N2).

**Table 2:** **Characteristics of laboratory-confirmed influenza cases aged 60+ years included in the analysis, influenza seasons 2010/11-2012/13, Germany**

	**2010/11 n (%)**	**2011/12 n (%)**	**2012/13 n (%)**
**Total**	1,174 (100)	767 (100)	5,217 (100)
**Cases vaccinated**	162 (14.0)	208 (27.1)	921 (17.7)
**Influenza subtypes**			
Influenza type A	774 (65.9)	537 (70.0)	3,069 (58.8)
Influenza type B	251 (21.4)	97 (12.7)	1,267 (24.3)
Influenza A/B^1^	137 (11.7)	89 (11.6)	806 (15.5)
No data	12 (1.0)	44 (5.8)	75 (1.4)
**Influenza strains**			
A(H1N1)pdm	582 (49.6)	12 (1.6)	859 (16.5)
A(H3N2)	1 (0.1)	118 (15.4)	168 (3.2)
B	251 (21.4)	97 (12.7)	1267 (24.3)
Not genotyped	137 (11.7)	89 (11.6)	806 (15.5)
Missing data on strains	203 (17.3)	451 (58.8)	2117 (40.6)
**Men (%)**	589 (50.2)	306 (40.0)	2440 (46.8)
**Age in years (mean ± SD)**	67.2 (±6.9)	74.7 (±9.9)	71.2 (±8.9)
**Age-group (years)**			
60-69	797 (67.9)	263 (34.3)	2485 (47.6)
70-79	307 (26.2)	267 (34.8)	1736 (33.3)
≥80	70 (6.0)	237 (30.9)	996 (19.1)
**Hospitalization status**			
Hospitalized	455 (38.8)	317 (41.5)	2060 (39.5)
Not hospitalized	709 (60.4)	446 (58.5)	3087 (59.2)

Data may not add up to 100% due to missing data.

^1^Reported as influenza, type A/B, not specified.

Overall, for 66.2% of cases information on the date of vaccination was available. Among these vaccinated cases, only four (2.4%), one (0.5%) and four (0.4%) in the season 2010/11, 2011/12 and 2012/13, respectively, were considered as non-vaccinated, since onset of disease occurred within 14 days after vaccination. Excluding these nine cases from analysis did not affect VE estimates (for details see online Additional file 1: Table S1). Approximately 40% of cases were notified as hospitalized in each season.

### Vaccine effectiveness

Table 3 provides data on VE estimates in individuals aged 60+ stratified by influenza subtypes, age-group and hospitalization status for all seasons. In the season 2010/11, VE was 76% (95% CI, 70-81%) for the predominantly circulating influenza A(H1N1)pdm and 84% (95% CI, 76-90%) for influenza B. VE increased with age for both subtypes and were higher in hospitalized compared to non-hospitalized patients. In the season 2011/12, VE was 87% (95% CI, 14-100%) for A(H1N1)pdm, −9% (95% CI, −59-26%) for influenza A(H3N2) and 74% (95% CI, 55-86%) for influenza B. VE estimates against influenza A(H3N2) decreased with age, and were higher among hospitalized compared to non-hospitalized cases. However, a statistically significant protective effect against laboratory confirmed A(H3N2) infection was not observed. In the season 2012/13, VE was 74% (95% CI, 69–79) for influenza A(H1N1)pdm, 39% (95% CI, 13-58%) for influenza A(H3N2) and 73% (95% CI, 68-77%) for influenza B. In this season, VE estimates against influenza A(H3N2) decreased with age and showed no protective effect in individuals aged 80 and older and among non-hospitalized cases. For the sensitivity analysis, in total 1,168 (99.4%), 664 (86.6%) and 5,182 (99.3%) cases were included when applying a narrower definition for the influenza seasons. In none of the seasons did VE estimates differed by more than 3% from those calculated in the primary analysis (data not shown). Further sensitivity analyses, e.g., assuming that influenza vaccination coverage (VC) among excluded cases was 50% or 200% of VC among included cases, or assuming that 50% of cases with missing information on the exact date of vaccination were regarded as non-vaccinated are shown in the online supplement (Additional file 1: Tables S3–S5).

**Table 3:** **Vaccine effectiveness (VE in %) against laboratory-confirmed influenza stratified by influenza-subtype, age-groups and hospitalization status, season 2010/11-2012/13, Germany**

**Age-group (years)**	**Season 2010/11**	**Season 2011/2012**	**Season 2012/2013**
**VE against influenza (all types)**	n = 1,174	n = 767	n = 5,217
60-69	72 (65–77)	59 (43–71)	65 (61–69)
70-79	82 (75–87)	56 (43–67)	63 (58–67)
≥80	86 (71–94)	40 (22–55)	60 (54–66)
≥60	80 (76–83)	49 (39–57)	64 (63–67)
≥60 non-hospitalized	77 (72–81)^1^	37 (22–49)^2^	63 (59–66)^1^
≥60 hospitalized	83 (78–88)^1^	62 (51–72)^2^	67 (63–71)^1^
**VE against influenza A**	n = 774	n = 537	n = 3,069
60-79	75 (69–80)	55 (43–65)	62 (58–66)
≥80	86 (64–96)	36 (11–54)	54 (44–62)
≥60	77 (72–81)	46 (35–56)	60 (57–64)
≥60 non-hospitalized	72 (64–79)^2^	32 (14–47)^2^	57 (52–62)^2^
≥60 hospitalized	82 (75–88)^2^	63 (49–74)^2^	66 (61–71)^2^
**VE against influenza A (H1N1)pdm**	n = 582	n = 12	n = 859
60-79	74 (67–79)	85 (−6-100)	74 (68–79)
≥80	91 (63–99)	NA^3^	64 (39–80)
≥60	76 (70–81)	87 (14–100)	74 (69–79)
≥60 non-hospitalized	68 (58–76)^2^	NA^3^	70 (62–77)^1^
≥60 hospitalized	83 (76–89)^2^	NA^3^	80 (72–86)^1^
**VE against influenza A (H3N2)**	n = 1	n = 118	n = 168
60-79	NA^3^	33 (−16-63)	47 (18–66)
≥80	NA^3^	−49 (−171-17)	22 (−49-60)
≥60	NA^3^	−9 (−59-26)	39 (13–58)
≥60 non-hospitalized	NA^3^	−48 (−132-5)^2^	27 (−8-51)^2^
≥60 hospitalized	NA^3^	56 (−1-83)^2^	73 (30–92)^2^
**VE against influenza B**	n = 251	n = 97	n = 1,267
60-79	83 (74–89)	73 (49–86)	74 (69–79)
≥80	89 (55–99)	80 (8–98)	66 (52–75)
≥60	84 (76–90)	73 (54–85)	73 (68–77)
≥60 non-hospitalized	82 (72–89)^1^	73 (42–89)^1^	75 (69–80)^1^
≥60 hospitalized	87 (73–95)^1^	72 (39–89)^1^	69 (61–77)^1^

^1^Not-hospitalized vs. hospitalized statistically not significant (p > 0.05).

^2^Not-hospitalized vs. hospitalized statistically significant (p ≤ 0.05).

^3^No/low number of cases.

### Factors associated with vaccine failure

In all three seasons, factors independently associated with vaccine failure were identified among influenza A cases only (Table 4). The odds for a breakthrough disease increased with age (p ≤0.05 for all three seasons). In addition, in all seasons vaccine failures was significantly less common among hospitalized compared to non-hospitalized influenza A cases. Finally, a longer time period since start of the influenza season increased the odds of a A(H3N2) breakthrough infection, but only in the season 2011/12.

**Table 4:** **Factors independently associated with seasonal influenza vaccine failures among cases aged 60 years and older, Germany, seasons 2010/11-2012/13**

**Season**	**Influenza-type**	**Variable/Category**	**Adjusted OR (95% CI)**	**p-value**
**2010/11**	**A(H1N1)**	Age in years	1.03 (1.00-1.07)	0.04
		Weeks since begin influenza season^1^	1.02 (0.94-1.11)	0.67^2^
		Hospitalization	0.45 (0.28-0.73)	<0.01
**2011/12**	**A(H3N2)**	Age in years	1.07 (1.03-1.12)	<0.01
		Weeks since begin influenza season^1^	1.12 (1.01-1.26)	0.03
		Hospitalization	0.23 (0.09-0.62)	<0.01
**2012/13**	**A(H1N1)**	Age in years	1.05 (1.02-1.08)	<0.01
		Weeks since begin influenza season^1^	1.02 (0.95-1.11)	0.49^2^
		Hospitalization	0.51 (0.33-0.80)	<0.01
	**A(H3N2)**	Age in years	1.06 (1.02-1.11)	<0.01
		Weeks since begin influenza season^1^	1.11 (0.98-1.26)	0.08^2^
		Hospitalization	0.35 (0.12-1.00)	0.05

OR, Odds ratio; 95% CI, 95% confidence interval; ns, not statistically significant.

^1^Influenza season defined as from week 40 of the previous year to week 20 in the following year.

^2^Removing this (non-significant) variable did not influence the final model.

### Comparison of study results with results from other observational studies

Our literature search yielded 141 records in PubMed. Of twenty-two potentially relevant studies, 15 met the inclusion criteria and an additional 2 studies [33,34] were identified through the meta-analysis of Darvishian et al. Finally, data from 17 studies provided enough data and were extracted (seven in 2010/11, four in 2011/12, and six in 2012/13) [17,33-48]. In all seasons and for all influenza-subtypes, VE estimates from our study were close or within the 95% CI of almost all identified TND studies (Table 5). Of particular interest, the included studies did not identify a protective effect against laboratory-confirmed influenza A(H3N2) infection in the 2011/12 season [39,41,49] and no or low VE for the 2012/13 season [35,37,44], similar to what we have observed in our analysis.

**Table 5:** **Vaccine effectiveness (VE) against influenza subtypes among the elderly: comparison of results from this present study with results from observational studies in Europe using the test-negative design, seasons 2010/11-2012/13**

**Country [ref]**	**Age-group**		**VE** ^**§**^ **in % (95% CI)**	**VE** ^**§**^ **in % (95% CI)**	**VE** ^**§**^ **in % (95% CI)**
	**(Years)**	**VE-analysis**	**A(H1N1)pdm**	**A(H3N2)**	**B**
**Season 2010/11**					
**This study**	≥60	Crude	76 (70–81)	-	84 (76–90)
		Adjusted	-	-	-
**Europe (I-MOVE)** ^1^ [38]	≥60	Crude	73 (48–86)	-	48 (−2-73)
		Adjusted	72 (27–90)	-	56 (−38-86)
**UK** [42]	≥65	Crude	-	-	-
		Adjusted	70 (0–85)^2^	-	65 (20–80)^2^
**Spain I** [40]	≥50	Crude	67 (27–85)^3^	-	-
		Adjusted	69 (0–91)^3^	-	-
**Spain II** [43]	≥60	Crude	60 (20–80)^4^	-	-
		Adjusted	59 (16–76)^4^	-	-
**Spain III** [45]	≥65	Crude	41 (−36-74)^5^	-	-
		Adjusted	-	-	-
**Spain IV** [46]	≥65	Crude	54 (−18-82)^6^		
		Adjusted	-		
**Germany** [17]	≥60	Crude	89 (1–99)^3^	-	-
		Adjusted	92 (−67-100)^3^	-	-
**Season 2011/12**					
**This study**	≥60	Crude	87 (14–100)	−9 (−59-26)	74 (55–86)
		Adjusted	-	-	-
**Europe (I-MOVE)** ^1^ [39]	≥60	Crude	-	6 (−41-38)	-
		Adjusted	-	15 (−33-46)	-
**UK** [41]	≥65	Crude	-	-	-
		Adjusted	-	48 (−50-82)	-
**Spain I** [49]	≥65	Crude	-	4 (−106-55)^7^	-
		Adjusted	-	19 (−146-73)^7^	-
**Spain II** ^8^ [47]	≥65	Crude	-	26 (−69-78)	-
		Adjusted	-	-	-
**Season 2012/13**					
**This study**	≥60	Crude	74 (69–79)	39 (13–58)	73 (68–77)
		Adjusted	-	-	-
**Europe (I-MOVE)** ^1^ [37]	≥60	Crude	59 (14–80)	37 (−13-65)	44 (9–66)
		Adjusted	-	-	-
**UK** [44]	≥65	Crude	-	-	-
		Adjusted		−14 (−206-57)	65 (18–85)
**Denmark** [35]	≥65	Crude	-	−19 (−52-6)	64 (17–84)
		Adjusted	-	−11 (−41-14)	69 (26–87)
**Lithuania** ^8^ [48]	≥60	Crude	92 (−55-100)^9^		
		Adjusted	-		
**Portugal** ^8^ [33]	≥60	Crude	80 (19–95)^10^	-	-
		Adjusted	-	-	-
**Multicenter** ^11^ [34]	≥65	Crude^12^	34 (−22-64)	46 (−16-75)	46 (23–63)
		Adjusted	13 (−68-55)	39 (−40-73)	41 (12–61)

VE, vaccine effectiveness.

^§^VE point estimates were rounded off to whole numbers.

^1^I-MOVE pooled data from other European countries; therefore, overlapping with some of the individual studies from the same season is likely.

^2^approx. VE since data for this age group were presented as figures only.

^3^VE against all influenza types; >80% of all subtypes identified were H1N1.

^4^VE against all-type influenza hospitalization; 76% of all subtypes identified were H1N1.

^5^VE against all-type hospitalization; 95% of all subtypes identified were H1N1.

^6^VE against all influenza types; 56% of all subtypes identified were H1N1, 41% were B.

^7^VE against all influenza cases; 92% of all subtypes identified were H3N2.

^8^Data extracted from the study of Darvishian et al. [31].

^9^VE against all influenza types; distribution among the whole study population: 41% H1N1, 40% B, 19% H3N2.

^10^VE against all influenza types; distribution among the whole study population: 53% H1N1, 43% B.

^11^VE against hospitalization; data collected in Spain, France and in the Russian Federation.

^12^adjusted for study site.

## Discussion

In this study we were able to show that among the elderly VE against influenza A(H1N1)pdm and B was moderate in all three seasons 2010/11-2012/13, whereas VE against influenza A(H3N2) was low in two consecutive seasons. These VE estimates were comparable to results provided by other observational studies in Europe that used the TND, suggesting that the screening method can be a suitable tool to generate useful influenza VE estimates at least for this particular age-group.

In studies applying the TND, patients with medically attended acute respiratory illness are recruited into the study and –if performed in an appropriate way- systematically tested for influenza virus. Those with laboratory-confirmed influenza infection are defined as cases and otherwise as controls. Since both groups are recruited in one process and both derived from the same population the TND has been found to be less susceptible to bias [19]. In the absence of RCTs, the TND has been advocated as a valid method to calculate almost unbiased influenza VE estimates [18,50] under a wide range of assumptions [51]. As with all observational studies adjusting for important confounders has still to be performed [18]. However, in particular for the age-group 60+ there are often only small differences between crude and adjusted point estimates as shown in most of the observational studies identified in our study [17,35,38,40,43].

The screening method has been described as a rapid and cheap tool to survey vaccine effectiveness, particularly when denominator data on individuals are not available [25,26]. For the screening method, only a random sample of cases is needed. However, accurate and age-group specific VC rates are crucial to produce valid VE estimates [25,52]. In our study, the random sample derived from the national communicable disease reporting system. Although this system collects data on laboratory-confirmed influenza infections nationwide, selection bias (e.g., enhanced testing for influenza among chronically ill patients), observer bias (e.g., vaccinated cases are less likely to be swabbed for influenza) or reporting bias (e.g., differences in notification behavior among physicians) cannot completely be ruled out. In addition, data on important confounders are not systematically reported in the system and adjusting for these variables was not possible. However, we probably reduced the impact of these shortcomings since we calculated VE for small age strata among elderly patients, for whom annual influenza vaccination is generally recommended in Germany. Since our results were rather similar to those from studies using the TND, it can be hypothesized that in this particular age-group adjustment for underlying medical conditions might not play such an important role. Furthermore, age-group specific VC rates were calculated in our study from a large database of health insurance claims covering the majority of the German population. The database has been shown to be a valid and representative instrument to describe VC in various age-groups in Germany [27,53] - a prerequisite for valid VE estimates when the screening method is applied [25].

Due to the large sample size we were able to assess subtype-specific VE even in different age-strata within the elderly population. In contrast, in observational studies using the TND estimation of strain-specific VE in the elderly is often not possible or limited by large CIs [17,35,36,42,54]. Furthermore, the assessment of VE against severe influenza using the TND is possible but would require additional study sites in hospitals and is often logistically challenging and resource-demanding. In view of these strengths and limitations, the screening method can be regarded as an useful method for the rapid assessment of crude influenza VE in the elderly when data on laboratory-confirmed influenza cases are available.

In our study, one of the major findings was that VE against influenza A(H3N2) was low in two consecutive seasons. This finding confirms the results from previous studies where no or only little protective effect against influenza A(H3N2) was observed [35,37,39,41,44,49]. Since antigenetic changes of the A/Victoria/361/2011(H3N2)-like vaccine during the manufacturing process has been reported, the WHO has recommended that the influenza A(H3N2) vaccine component for use in the 2013/14 season should be updated [55].

In addition to older age, the probability of vaccine failure increased over time during season 2011/12 among cases infected with influenza A(H3N2). The same effect has been observed in other settings and it was discussed that this may be due to virus changes or waning immunity after vaccination [39,41,49]. Due to the higher mutation rate of influenza A compared to B viruses [56] it is plausible that these effects are mainly identified among patients infected with influenza A.

Interestingly, we found that vaccine failure was negatively associated with hospitalization. This was true in all seasons among cases infected with influenza A viruses. A recently conducted study in Spain suggested that VE may be greater in preventing severe than in preventing mild cases, and that the benefits of vaccination may be greater than suggested [57]. However, in our study we cannot fully exclude that this effect was due to bias, e.g. the healthy vaccinee effect or different testing behavior for influenza in vaccinated inpatients and vaccinated outpatients. Further studies exploring these potential VE differences by clinical outcome would be important to better estimate the true effect of influenza vaccination in preventing severe influenza and influenza-related mortality.

### Strengths and limitations

Our study benefits from its large sample size. During three consecutive influenza seasons we were able to include more than 7,000 laboratory-confirmed influenza cases aged 60+ both from the outpatient and inpatient sector. Due to this large size we were able to assess subtype-specific VE even in age-strata of those aged 80+. In addition, since we used age-specific VC data for each single season derived from a large database representing ~85% of the German population, VC can be regarded as representative. Finally, by limiting the studies to individuals aged 60+ we were probably able to reduce the risk of biases to at least some extent, which was reflected by similar VE estimates when comparing to studies using the TND.

However, some limitations have to be acknowledged. First, cases were identified by the mandatory disease surveillance system and the quality of our analysis depends on the quality of the reported data transmitted to the RKI. Since only laboratory-confirmed infections are notifiable in Germany and since it is the decision of the physician whether or not to swab patients for influenza, we cannot exclude that vaccinated persons were less likely be tested for influenza virus infection, which might have led to an overestimation of VE. However, this would not explain differences in VE between hospitalized and non-hospitalized cases. Second, cases were defined as vaccinated if they were notified as “currently vaccinated” and considered as non-vaccinated if disease onset occurred within 14 days after vaccination. Since date of vaccination was provided by 66% of all cases only, it is possible that in cases with unknown vaccination date disease occurred within 14 days after receipt of the vaccine leading to an underestimation of VE. However, since the proportion among cases with information on vaccination date with disease onset prior 14 days after receipt of vaccine was less than 1%, it seems rather unlikely that this proportion was significantly higher among those without information on vaccination date. Third, we were not able to control for some important confounders such as comorbidities, but limiting our study population to persons aged 60+ might have accounted for this. Fourth, although >85% of all cases were laboratory confirmed using PCR or cell-culture, subtyping was only performed in 70% of cases. Finally, since vaccine types are not reported systematically for notified influenza cases, vaccine type or product specific VE cannot be calculated. Overall, optimizing the completeness of the data regarding hospitalization status, vaccination status, and date of vaccination as well as additional information on comorbidities could improve the validity of our VE estimates.

## Conclusions

In conclusion, the screening method can be used to rapidly assess influenza subtype-specific VE among the elderly in Germany. Due to high numbers of cases, VE can be estimated even for rather narrow age-ranges and non-dominating influenza subtypes within the elderly population as well as for hospitalized and non-hospitalized patients. Since adjusting for important confounders and the assessment of product-specific VE is not possible, the screening method can be regarded as an important but only supplementary tool for assessing crude VE, in addition to more precise but also more resource-demanding TND-studies for the post-marketing evaluation of influenza vaccines.

## Additional file

Additional file 1: Table S1. Sensitivity analysis in which cases (n=9) were excluded who were vaccinated <14 days prior to symptom onset. **Table S2.** Baseline characteristics of excluded vs. included cases. **Table S3.** Sensitivity analysis in which vaccination coverage among excluded cases was considered 50% and 200% of vaccination coverage of included cases, respectively. **Table S4.** Baseline characteristics of cases with exact date of vaccination compared to those without this information. **Table S5.** Sensitivity analysis in which 50% of cases without information on exact date of vaccination were considered as non-vaccinated.

## Abbreviations

ARI: Acute respiratory illness
ASHIP: Association of Statutory Health Insurance Physicians
CI: Confidence interval
NITAG: National Immunization Technical Advisory Group
PCV: Proportion cases vaccinated
PV: Proportion population vaccinated
OR: Odds ratio
RCT: Randomized controlled trial
RKI: Robert Koch Institute
TND: Test-negative design
VC: Vaccine coverage
VE: Vaccine effectiveness
