# Supplementary material for: Application of the screening method to monitor influenza vaccine effectiveness among the elderly in Germany
Source: BMC Infect Dis. 2015 Mar 20;15:137. doi: 10.1186/s12879-015-0882-3 (PMC4371628; doi:10.1186/s12879-015-0882-3)
Supplement: Additional file 1: Table S1. — Sensitivity analysis in which cases (n=9) were excluded who were vaccinated <14 days prior to symptom onset. Table S2. Baseline characteristics of excluded vs. included cases. Table S3. Sensitivity analysis in which vaccination coverage among excluded cases was considered 50% and 200% of vaccination coverage of included cases, respectively. Table S4. Baseline characteristics of cases with exact date of vaccination compared to those without this information. Table S5. Sensitivity analysis in which 50% of cases without information on exact date of vaccination were considered as non-vaccinated. [file 12879_2015_882_MOESM1_ESM.docx]

**Additional file 1**

to the manuscript entitled “Application of the screening method to monitor influenza vaccine effectiveness among the elderly in Germany” by Cornelius Remschmidt et al.

**Table S1**. Sensitivity analysis in which cases (n=9) were excluded who were vaccinated <14 days prior to symptom onset compared to the analysis presented in the paper. All cases were aged between 60 and 79 years.

|  |  |  |  |  |
| --- | --- | --- | --- | --- |
| **Season** | **Influenza-type** | **Age-group** | **Original analysis*^1^  (VE in %)** | **Sensitivity analysis*^2^  (VE in %)** |
| 2010/11 | Influenza A | 60-79 | 75 (69-80) | 75 (69-80) |
|  | Influenza B | 60-79 | 83 (74-89) | 82 (73-89) |
| 2011/12 | Influenza B | 60-79 | 73 (49-86) | 72 (49-86) |
| 2012/13 | Influenza A | 60-79 | 62 (58-66) | 62 (58-66) |
| *^1^ cases who were vaccinated <14 days prior symptom onset were considered as non-vaccinated  *^2^ cases who were vaccinated <14 days prior symptom onset were excluded | | | | |

**Table S2**. Baseline characteristics of excluded vs. included cases, Germany, 2010/11-2012/13.

|  |  |  |  |  |
| --- | --- | --- | --- | --- |
|  | | **Included cases** | **Excluded cases** | **p-value*** |
| **Season 2010/11** |  | N=1,174 n (%) | N=197 n (%) |  |
| Sex | Male | 589 (59) | 111 (56) | 0.11 |
| Age group | 60-69 | 797 (68) | 135 (69) | 0.97 |
|  | 70-70 | 307 (26) | 51 (26) |  |
|  | ≥ 80 | 70 (6) | 11 (6) |  |
| Influenza strain | A(H1N1)pdm | 582 (60) | 106 (64) | 0.16 |
|  | A(H3N2) | 1 (<1) | 1 (1) |  |
|  | B | 251 (26) | 44 (27) |  |
|  | not subtyped | 137 (14) | 15 (9) |  |
| Hospitalized | Yes | 455 (39) | 81 (41) | 0.59 |
| **Season 11/12** |  | N=767 n (%) | N=266 n (%) | **p-value*** |
| Sex | Male | 306 (40) | 101 (38) | 0.6 |
| Age group | 60-69 | 263 (34) | 71 (27) | <0.01 |
|  | 70-70 | 268 (35) | 73 (27) |  |
|  | ≥ 80 | 237 (31) | 122 (46) |  |
| Influenza strain | A(H1N1)pdm | 12 (4) | 4 (6) | 0.25 |
|  | A(H3N2) | 118 (37) | 35 (49) |  |
|  | B | 97 (31) | 18 (25) |  |
|  | not subtyped | 89 (28) | 15 (21) |  |
| Hospitalized | Yes | 317 (41) | 115 (43) | 0.59 |
| **Season 12/13** |  | N=5,217 n (%) | N=1,586 n (%) | **p-value*** |
| Sex | Male | 2,440 (47) | 778 (49) | 0.11 |
| Age group | 60-69 | 2,485 (48) | 648 (41) | <0.01 |
|  | 70-70 | 1,736 (33) | 518 (33) |  |
|  | ≥ 80 | 996 (19) | 420 (26) |  |
| Influenza strain | A(H1N1)pdm | 859 (28) | 252 (30) | 0.31 |
|  | A(H3N2) | 168 (5) | 36 (4) |  |
|  | B | 1,267 (41) | 332 (39) |  |
|  | not subtyped | 806 (26) | 231 (27) |  |
| Hospitalized | Yes | 2,060 (40) | 679 (44) | 0.01 |
| * p-values for the chi-squared test | |  |  |  |

**Table S3**. Sensitivity analysis in which vaccination coverage among excluded cases was considered 50% and 200% of vaccination coverage of included cases, respectively, compared to the analysis presented in the paper (“original analysis”).

|  |  |  |  |
| --- | --- | --- | --- |
| Age group (years) | **Original analysis**  VE (%) | VC among excluded cases considered **50%** of VC of included cases VE (%) | VC among excluded cases considered **200%** of VC of included cases VE (%) |
| **Season 2010/11** | 162/1,074 vacc. | 176/1,371 vacc. | 216/1,371 vacc. |
|  |  |  |  |
| ≥ 60 | 80 (76-83) | 81 (78-84) | 76 (72-79) |
| **Season 11/12** | 208/767 vacc. | 244/765 vacc. | 353/767 vacc. |
| ≥ 60 | 49 (39-57) | 57 (51-63) | 28 (19-37) |
| **Season 12/13** | 921/5,217 vacc. | 1,061/6,803 vacc. | 1,479/6,803 vacc. |
| ≥ 60 | 64 (63-67) | 69 (67-71) | 54 (51-56) |
| VE, vaccine effectiveness; VC, vaccination coverage; vacc., vaccinated | | | |

**Table S4**. Baseline characteristics of cases with exact date of vaccination vs. those without this information; Germany, 2010/11-2012/13.

|  |  |  |  |  |
| --- | --- | --- | --- | --- |
|  | | **Cases with information on vaccination date** | **Cases without information on vaccination date** | **p-value*** |
| **Season 2010/11** |  | n/N=122  (%) | n/N=40  (%) |  |
| Sex | Male | 56 (46) | 19 (48) | 0.86 |
| Age group | 60-69 | 83 (68) | 26 (65) | 0.89 |
|  | 70-70 | 32 (26) | 12 (30) |  |
|  | ≥ 80 | 7 (6) | 2 (5) |  |
| Influenza strain | A(H1N1)pdm | 69 (70) | 23 (62) | 0.14 |
|  | A(H3N2) | 1 (1) | 0 |  |
|  | B | 16 (16) | 12 (32) |  |
|  | not subtyped | 12 (13) | 2 (5) |  |
| Hospitalized | Yes | 36 (30) | 17 (43) | 0.13 |
| **Season 11/12** |  | n/N=137  (%) | n/N=70  (%) | **p-value*** |
| Sex | Male | 64 (47) | 20 (29) | 0.01 |
| Age group | 60-69 | 34 (24) | 10 (14) | 0.13 |
|  | 70-70 | 43 (32) | 30 (43) |  |
|  | ≥ 80 | 60 (44) | 30 (43) |  |
| Influenza strain | A(H1N1)pdm | 1 (2) | 0 | 0.76 |
|  | A(H3N2) | 31 (54) | 21 (64) |  |
|  | B | 10 (18) | 5 (15) |  |
|  | not subtyped | 15 (26) | 7 (21) |  |
| Hospitalized | Yes | 49 (36) | 18 (28) | 0.14 |
| **Season 12/13** |  | n/N=612  (%) | n/N=305  (%) | **p-value*** |
| Sex | Male | 274 (45) | 124 (41) | 0.24 |
| Age group | 60-69 | 204 (33) | 93 (30) | 0.63 |
|  | 70-70 | 241 (39) | 129 (43) |  |
|  | ≥ 80 | 167 (27) | 83 (27) |  |
| Influenza strain | A(H1N1)pdm | 73 (26) | 41 (23) | 0.01 |
|  | A(H3N2) | 17 (6) | 28 (16) |  |
|  | B | 112 (40) | 66 (37) |  |
|  | not subtyped | 80 (28) | 42 (24) |  |
| Hospitalized | Yes | 219 (36) | 120 (40) | 0.27 |
| * p-values for the chi-squared test | |  |  |  |

**Table S5**. Sensitivity analysis in which 50% of cases without information on exact date of vaccination were considered as non-vaccinated. Vaccine effectiveness (%) against all influenza types.

| **Age group (years)** | **Original analysis** assuming that all  cases without information on exact date of vaccination were vaccinated VE (%) | **Sensitivity analysis** assuming that  50% of cases without information on exact date of vaccination were non-vaccinated VE (%) |
| --- | --- | --- |
| **Season 2010/11** | 162 vacc./1,022 non-vacc. | 142 vacc./1,042 non-vacc. |
| All age groups ≥ 60 | 80 (76-83) | 83 (65-73) |
| **Season 11/12** | 208 vacc./559 non-vacc. | 173 vacc./594 non-vacc. |
| All age groups ≥ 60 | 49 (39-57) | 60 (53-66) |
| **Season 12/13** | 921 vacc./4,296 non-vacc. | 769 vacc./4,448 non-vacc. |
| All age groups ≥ 60 | 64 (63-67) | 71 (69-73) |
| VE, vaccine effectiveness; vacc., vaccinated | |  |
